# The Profile of Secondary Metabolites and Other Bioactive Compounds in *Cucurbita pepo* L. and *Cucurbita moschata* Pumpkin Cultivars

**DOI:** 10.3390/molecules24162945

**Published:** 2019-08-14

**Authors:** Bartosz Kulczyński, Anna Gramza-Michałowska

**Affiliations:** Department of Gastronomy Sciences and Functional Foods, Faculty of Food Science and Nutrition, Poznań University of Life Sciences, Wojska Polskiego 31, 60–624 Poznań, Poland

**Keywords:** pumpkin, *Cucurbita pepo* L., *Cucurbita moschata* Duchesne ex Poir, phytonutrients, polyphenols, carotenoids

## Abstract

Plants and animals are sources of various bioactive compounds that exhibit a broad spectrum of health-promoting effects. Scientists continue studies on the chemical composition of many products in search of foods with high nutritional value. The pumpkin (*Cucurbita* sp.) is unquestionably a source of valuable nutrients. This vegetable is well-known all over the world and it is appreciated due to its high content of carotenoids, but it is still not much used in the processing industry. The aim of present study was to compare the flesh of 15 pumpkin varieties belonging to the *Cucurbita pepo* and *C. moschata* species in terms of the bioactive compound content (carotenoids, phenolic acids, flavonols, minerals and vitamins) and to demonstrate whether the variety has an effect on the chemical composition. To date, no such extensive research has been carried out in this area. The research revealed that the pumpkin pulp had high content of carotenoids. In nearly all cases lutein was the most abundant carotenoid. Numerous phenolic acids and flavonols were also identified. All the cultivars contained gallic acid, protocatechuic acid, 4-hydroxybenzoic acid, vanillic acid, chlorogenic acid, caffeic acid, and rutin. The pumpkin pulp also contained alpha- and gamma-tocopherol. No beta- or delta-tocopherol was found. Potassium, calcium, and sodium were the most abundant minerals. The research also proved that the profile of bioactive compounds in the pumpkin pulp was considerably diversified and depended on the species and cultivar

## 1. Introduction

Bioactive compounds are an area of dieticians and food technologists’ interest. They are used for the production of enriched food with enhanced health-promoting properties, which is an important element of the human diet. Food manufacturers are increasingly willing to use bioactive compounds to enhance the health-promoting properties of food products. An example of such food are yoghurts with inulin, chlorella- or spirulina-enriched bars, eggs enriched with omega-3 fatty acids, margarines with the addition of plant sterols, and lycopene drinks. Increasingly popular are dietary supplements with compositions based on plant extracts that are sources of bioactive compounds (e.g., milk thistle—silymarin, black pepper—piperine, green coffee—chlorogenic acid, ginkgo biloba leaves—ginkgo flavone glycosides, green tea—epigallocatechin gallate, ashwagandha—withanolides, grape skins—trans-resveratrol). Bioactive compounds include components derived from plants and animals, which are beneficial to health when consumed [1,2]. The group of bioactive compounds chiefly includes the following components: vitamins, minerals, terpenes, polyphenols, carotenoids, prebiotics, probiotics, soluble and insoluble dietary fiber, stanols, sterols, amino acids, bioactive proteins, peptides, and fatty acids. They exhibit a broad spectrum of health-promoting effects, i.e., cardioprotective, hypoglycaemic, antioxidative, anti-cancer, anti-bacterial, immunomodulatory, neuroprotective and anti-inflammatory effects [3,4,5,6,7,8,9,10]. In recent years, research on the content of bioactive compounds not only in terms of “popular” products, such as chia seeds, chlorella, moringa oleifera etc. [11,12,13] or fruits of various plants [14,15,16] has been intensively carried out. It has been documented many times that also extracts of other organs of plants, such as bark, roots, and leaves, can be a source of bioactive compounds with confirmed health properties, e.g., polyphenols [17,18]. In addition, the content of bioactive compounds, including antioxidants is increasingly analyzed in plants that are not widely known [19]. This allows for the discovery of new plant raw materials, which in the future may be a part of the diet as a source of nutrients important for human health. It is worth noting that bioactive compounds not only affect human health but are also used in food technology. Due to the high antioxidant potential, these compounds prevent lipid oxidation (e.g., in meat products) and, as a result, prolong the shelf life of food products. An example is ascorbic acid (E300) and tocopherols (E306-309) [20,21]. The bioactive compounds may also inhibit the enzymatic browning processes by affecting the color retention of products [22]. The plant extracts that are the source of active compounds are attributed to antimicrobial activity and, therefore, may be potential preservatives [23].

Pumpkin (*Cucurbita* L.) is a source of various phytochemicals with documented health-promoting properties. It belongs to the *Cucurbitaceae* family, which comprises about 130 species both growing in the wild and cultivated all over the world. There are about 20 species belonging to the pumpkin (*Cucurbita*) genus. *Cucurbita maxima*, *C. pepo* L., *C. moschata* Duchesne ex Poir, *C. fificolia*, and *C. argyrosperma* are the most commonly cultivated pumpkin species. All anatomical parts of the plant are edible, but seeds and pulp are particularly important for food processing and nutrition [24,25]. In the food industry, pumpkin pulp is mostly used for the production of purées, dishes, and juices for children and babies. Candied pumpkin pulp, dried snacks, frozen products, jams, and marinades are also prepared from pumpkin pulp. The use of pumpkin pulp [26,27,28,29] as a natural pigment in the form of powder added to confectionery, bakery, pasta, and dairy products is a new trend in processing. To date, no studies have been carried out in which the influence of pumpkin-based product consumption on human health has been analyzed. There are studies that focus on the health properties of pumpkin seed oil. Other studies involving animals have shown that pumpkin seed oil protects against the genotoxicity caused by bisphenol A and azathioprine [30,31]. It was also found that pumpkin seed oil is characterized by antihypertensive and cardioprotective effects. The mechanism responsible for this effect is probably due to the effect of pumpkin seed oil on NO (nitric oxide) production [32]. It is also believed that pumpkin seed oil may be effective in the treatment of benign prostatic hyperplasia [33]. The chemical composition of pumpkin pulp is considerably diversified and it depends both on the species and variety. It has low content of proteins, fats, and carbohydrates. The pumpkin is a low-calorie, easily digestible vegetable. The energy value of its pulp amounts to about 30 kcal/100 g [34]. Pumpkins with orange pulp are particularly valuable, because they have a high content of carotenoids, especially beta-carotene and lutein [35,36,37].

Thus far there have not been many studies comparing the species- and variety-dependent profile of individual carotenoids. Kreck et al. showed that pumpkin varieties belonging to *Cucurbita maxima* differ significantly in terms of carotenoids. The author also showed that in the tested pumpkin varieties the concentration of lutein was in the range of 20–146 mg/kg dry mass, however, in the ‘Muskat’, ‘Baby Bear’, and ‘Butternut’ no presence of this compound was found. Similarly, for beta-carotene (17–263 mg/kg dry mass), statistically significant differences were also found [38]. In addition, Biesiada et al. also observed that the total carotenoids content in pumpkin fruit varies in a wide range (0.57–18.40 mg/100 g fresh mass) [39]. Additionally, reference publications lack data on the content of other bioactive compounds contained in pumpkin pulp.

The aim of this study was to compare the content of bioactive compounds: carotenoids, polyphenols, flavonoids, tocopherols, minerals, vitamin C, B1, and folates in 15 cultivars of two pumpkin species, *Cucurbita pepo* L. and *Cucurbita moschata* Duchesne ex Poir.

## 2. Results and Discussion

### 2.1. Moisture Content

The water content in the tested pumpkin varieties ranged from 82.35 to 95.16% (Table 1). The highest water content was in the following varieties: ‘Miranda’, ‘Snow Ball’, ‘Spaghetti’, and ‘Yuxijiangbinggua’. The lowest water content was found for ‘Delicata’, ‘Futsu’, and ‘Baby Boo’.

### 2.2. Carotenoid Content in C. pepo L. and C. moschata Duchesne ex Poir

The analysis of the content of carotenoids showed high variability among the pumpkin cultivars (Table 1). All the cultivars belonging to the *C.pepo* L. and *C. moschata* Duchesne ex Poir species contained zeaxanthin, lutein, and beta-carotene. The highest content of zeaxanthin was found in the ‘Festival’, ‘Orangita’, and ‘Casperita’ cultivars. The lowest concentration of zeaxanthin was measured in ‘Shishigatani’, ‘Butternut’, and ‘Butterkin’. The following cultivars were the richest sources of lutein: ‘Table Queen’, ‘Orangita’, and ‘Spaghetti’. The ‘Delicata and ‘Miranda cultivars also contained large amounts of lutein. The lowest contents of lutein were measured in ‘Orange Butternut’, ‘Shishigatani’, and ‘Butterkin’. The highest contents of beta-carotene were found in the ‘Table Queen’ and ‘Baby Boo’ cultivars. They were also high in ‘Delicata’ and ‘Orange Butternut’. The results of measurements of the content of the three carotenoids enabled us to calculate the retinol equivalent (RE). The highest RE values were observed in the following cultivars: ‘Orangita’, ‘Table Queen’, ‘Baby Boo’, and ‘Delicata’. The lowest RE values were found in ‘Shishigatani’ and ‘Butterkin’.

Jaswir et al. [40] measured the following contents of zeaxanthin, lutein and beta-carotene in pumpkin of the *Cucurbita moschata* Duchesne ex Poir species: 2.26 µg/g (zeaxanthin), 425.3 µg/g (lutein), 1516.5 µg/g (beta-carotene). In our study the content of zeaxanthin in all the pumpkins was higher, on the other hand, the pumpkin analyzed by Jaswir et al. contained much more lutein and beta-carotene [41]. Researchers also observed that the content of carotenoids depended on the harvest period as well as storage period. Jaswir et al. showed that the highest lutein content was present in the pumpkins harvested in June, while the largest amounts of beta-carotene were observed in pumpkins harvested in February. The authors also observed that the carotenoids content increased in the second month of storage. The content of lutein and beta-carotene was reduced during the 3rd and 4th month of storage. In the fifth month of storage, the content of lutein and beta-carotene increased. In the sixth month there was an increase in the lutein content, while the concentration of beta-carotene was reduced. In the case of zeaxanthin, its content alternately increased and decreased every month during six months of storage. In our study, the pumpkin was stored for about 2–3 months, which in relation to the mentioned research results was the optimal period in terms of the concentration of these compounds. All pumpkins were bought at the same time and were stored for the same time. However, the subject of our study was not to determine the effect of pumpkin storage on the content of bioactive compounds. Biesiada et al. [39] also confirmed the influence of storage on the content of carotenoids. They noted the highest concentrations of total carotenoids in the ‘Amazon’ and ‘Ambar’ cultivars. After storage they were reduced to 11.69 and 7.42 mg/100 g, respectively. Carvalho et al. [42] measured the content of alpha- and beta-carotene in pumpkin (*Cucurbita moschata* Duchesne ex Poir) and it amounted to 39.95 and 172.20 μg/g, respectively. All the cultivars investigated in our study had lower content of beta-carotene. Apart from that, the authors found the presence of isomers: 9-beta-carotene (0.86 μg/g) and 13-beta-carotene (3.64 μg/g). In addition, thermal treatment had influence on the content of carotenoids. The investigations showed that cooking in water and steaming increased the concentration of carotenoids. Zdunic et al. [43] analyzed the content of carotenoids in pumpkin pulp and its products. They found that the total amount of these compounds in unprocessed pumpkin was 86.3 µg/g. At the same time, the authors observed a decrease in the concentration of carotenoids in the pumpkin products jam (63.9 μg/g) and juice (28.6 μg/g). Provesi, Diaz and Amante [44] noted a difference in the content of total carotenoids in cultivars of two pumpkin species: *C. moschata* Duchesne ex Poir (‘Menina Brasileira’) and *C. maxima* (‘Exposicao’). The pumpkin of the *C. maxima* species contained less carotenoids. Kreck et al. analyzed the profile of carotenoids in seven pumpkin cultivars: ‘Muskat’, ‘Bischofsmutze’, ‘Baby Bear’, ‘Butternut’, ‘Rouge’, ‘Neon’, and ‘Hokkaido’ (*C. maxima*) [38]. The highest content of beta-carotene was found in the ‘Muskat’ cultivar. This cultivar contained more beta-carotene than all the cultivars analyzed in our study. On the other hand, the content of beta-carotene in the other cultivars researched by Kreck et al. (17–132 mg/kg) was similar to the results obtained in our experiment (1.29–8.33 mg/100 g) [38]. As far as lutein is concerned, the content of this compound in the cultivars analyzed in our experiment (3.34–22.92 mg/100 g) was similar to the cited results (20–146 mg/kg). The authors found zeaxanthin only in one cultivar, i.e., ‘Rouge’. Its concentration was similar to the content of this compound in the cultivars analyzed in our study, i.e., ‘Spaghetti’ (0.61 mg/100 g) and ‘Miranda’ (0.86 mg/100 g). There were also other carotenoids: alpha-carotene, alpha- and beta-cryptoxanthin, 9-cis-beta-carotene, 13-cis-beta-carotene, luteoxanthin, violaxanthin, and neoxanthin. However, these compounds were not identified in all the cultivars. Our results are limited to investigate the contents of the three types of carotenoids that are most commonly discussed, and which presence (based on literature data) can be expected in the pumpkin flesh. However, it is possible that a certain amount of, e.g., alpha-carotene or lycopene could also be found in the pumpkin fruit.

### 2.3. Polyphenolic Compounds Content in C. pepo L. and C. moschata Duchesne ex Poir

In this work, it was decided to determine the content of selected phenolic compounds. We have chosen phenolic acids and flavonols which are most commonly found in plant-derived products, and which are also most often discussed in the scientific literature. Many phenolic acids were identified in the pumpkin cultivars in our study (Table 2). The highest contents of gallic acid were measured in the following cultivars: ‘Shishigatani’, ‘Casperita’, and ‘Table Queen’. There were small amounts of this compound in ‘Butterkin’, ‘Yuxijiangbinggua’, and ‘Festival’. The following cultivars had high content of protocatechuic acid: ‘Shishigatani’, ‘Delicata’, and ‘Orange Butternut’. As in the case of gallic acid, the ‘Festival’ cultivar had small concentration of protocatechuic acid. Additionally, there were low levels of this compound in ‘Snow Ball’ and ‘Casperita’. The highest content of 4-hydroxybenzoic acid was found in the ‘Yuxijiangbinggua’ cultivar. There were also high levels of this acid in ‘Butternut’ and ‘Table Queen’. There were small amounts of vanillic acid found in the pumpkin cultivars in our study. The highest contents were measured in ‘Shishigatani’ and ‘Snow Ball’, whereas the lowest concentrations were observed in ‘Spaghetti’, ‘Baby Boo’, and ‘Orange Butternut’. The results showed that the cultivars of both pumpkin species were characterized by low content of chlorogenic acid. Its highest levels were observed in ‘Casperita’ and ‘Orange Butternut’, whereas the lowest levels were found in ‘Butterkin’, ‘Futsu’, and ‘Baby Boo’. Many pumpkin cultivars had high concentrations of caffeic acid. ‘Orangita’, ‘Miranda’, and ‘Spaghetti’ were the richest sources of this acid, whereas ‘Butternut’, ‘Butterkin’, and ‘Shishigatani’ had the lowest amounts of this compound. ‘Miranda’ and ‘Table Queen’ were the richest sources of ferulic acid, whereas ‘Spaghetti’, ‘Butterkin’, and ‘Snow Ball’ had the lowest concentrations of this compound. The largest amounts of sinapic acid were found in ‘Butternut’, ‘Table Queen’, and ‘Orange Butternut’.

The content of flavonols was also analyzed (Table 3). The concentrations of flavonols were lower than the content of phenolic acids. Rutin and kaempferol were the most abundant flavonols. Among the cultivars under study, ‘Butternut’ and ‘Snow Ball’ were the richest sources of rutin. The highest contents of kaempferol were found in ‘Festival’, ‘Table Queen’, and ‘Orange Butternut’. There was no kaempferol in ‘Miranda’, ‘Snow Ball’, ‘Shishigatani’, ‘Futsu’, ‘Casperita’, ‘Baby Boo’, or ‘Delicata’. There were small amounts of isoquercetin in ‘Orange Butternut’ and ‘Delicata’. The compound was absent from ‘Spaghetti’, ‘Miranda’, ‘Shishigatani’, ‘Butterkin’, ‘Futsu’, ‘Yuxijiangbinggua’, ‘Baby Boo’, and ‘Table Queen’. The highest contents of astragalin were found in ‘Orange Butternut’ and ‘Orangita’, but the compound was absent from six cultivars: ‘Miranda’. ‘Butternut’, ‘Shishigatani’, ‘Yuxijiangbinggua’, ‘Casperita’, and ‘Delicata’. The highest contents of myricetin were found in ‘Baby Boo’ and ‘Orangita’, but the compound was absent from ‘Snow Ball’, ‘Butternut’, ‘Shishigatani’, ‘Orange Butternut’, ‘Casperita’, and ‘Festival’. The research showed that the highest concentrations of quercetin were found in ‘Snow Ball’, ‘Casperita’ and ‘Baby Boo’. There was no quercetin in the following cultivars ‘Spaghetti’, ‘Miranda’, ‘Shishigatani’, ‘Butterkin’, ‘Futsu’, ‘Orange Butternut’, and ‘Delicata’.

Iswaldi et al. found a large number of polyphenolic compounds in three pumpkin cultivars (‘Verde’, ‘Redondo’, and ‘Organic Verde’) of the *Cucurbita pepo* species [45]. As far as hydroxycinnamic acids and their derivatives are concerned, all the cultivars contained p-coumaric acid and ferulic acid. The results of our research showed that ferulic acid was present in all the cultivars except ‘Shishigatami’. On the other hand, we did not find p-coumaric acid in six cultivars: ‘Snow Ball’, ‘Butterkin’, ‘Futsu’, ‘Casperita’, ‘Delicata’, and ‘Table Queen’. Researchers observed chlorogenic and caffeic acids only in the ‘Organic Verde’ cultivar [46]. We found these compounds in all the cultivars analyzed in our study. The authors also found the following components: caftaric acid, 2-O-caffeoylmalic acid, chicoric acid, dicaffeic acid, and sinapic acid hexoside. Apart from that, they found p-hydroxybenzaldehyde, benzoic acid, vanillic acid glycoside, hydroxybenzoic acid hexose, and 3,4,5 tri-O-galloylquinic acid in two cultivars, i.e., ‘Verde’ and ‘Redondo’. [46]. Priori et al. [47] measured the total content of polyphenols in various pumpkin cultivars (*Cucurbita moschata* Duchesne ex Poir). It ranged from 26.3 to 79.9 mg/100 g. Dragovic-Uzelac et al. [48] compared the content of phenolic compounds (chlorogenic acid, caffeic acid, p-coumaric acid, syringic acid) in three pumpkin species: *C. maxima*, *C. pepo* L. and *C. moschata* Duchesne ex Poir. The highest content of chlorogenic acid was found in the ‘Turkinja/II’ cultivar (*C. maxima*; 21.34 mg/kg). The content of caffeic acid amounted to 1.15 mg/kg in the ‘Argenta/I’ (*C. moschata* Duchesne ex Poir) and ‘Turkinja/I’ cultivars (*C. maxima*). Apart from that, p-coumaric acid was identified only in the ‘Argenta/I’ cultivar (1.05 mg/kg). The highest amount of syringic acid was found in ‘Argenta/I’ (25.09 mg/kg) and ‘TableGold/II’ (*C. pepo* L., 22.11 mg/kg).

### 2.4. Tocopherols Content in C. pepo L. and C. moschata Duchesne ex Poir

There were two forms of tocopherols in the pumpkin cultivars under analysis: alpha- and gamma-tocopherols (Table 4). None of the cultivars contained beta- or delta-tocopherols. The highest contents of alpha-tocopherol were found in ‘Spaghetti’ and ‘Casperita’. They were also relatively high in ‘Yuxijiangbinggua’ and ‘Baby Boo’. The lowest contents of alpha-tocopherol were found in ‘Snow Ball’, ‘Festival’, and ‘Shishigatani’. The following cultivars had high content of gamma-tocopherol: ‘Orange Butternut’, ‘Spaghetti’, and ‘Yuxijiangbinggua’. The lowest contents of gamma-tocopherol were found in ‘Butternut’, ‘Miranda’, and ‘Shishigatani’. The results let us calculate the alpha-tocopherol equivalent (alpha TE) for individual pumpkin cultivars. The highest alpha TE values were noted for ‘Spaghetti’, ‘Casperita’ and ‘Yuxijiangbinggua’, whereas the lowest values were noted for ‘Shishigatani’, ‘Festival’, and ‘Snow Ball’. So far there have been few studies on the content of tocopherols in pumpkin pulp. Seleim, Ali and Hassan (2015) noted the highest content of alpha-tocopherol in the ‘El-Zarka’ cultivar (*C. maxima*; 1.54 mg/100 g). It was similar to the content of alpha-tocopherol in the following cultivars: ‘Snow Ball’, ‘Festival’, and ‘Shishigatani’. The other cultivars in our study had higher content of this compound. The authors noted the lowest content of alpha-tocopherol in the ‘Faraskour’ cultivar (*C. maxima*; 774.52 µg/100 g). Procida et al. [49] analyzed the content of alpha- and gamma-tocopherols in various commercially available pumpkin seed oils. They found the highest concentrations of tocopherols in ‘Jerusalem’ (87.5 mg/100 g), ‘Fram’ (8.74 mg/100 g), and ‘Zvezda’ oils (65.4 mg/100 g), whereas it was the lowest in ‘Crudigno’ oil (18.1 mg/100 g). Petkova and Antova [50] studied the content of tocopherols in pumpkin seeds (*Cucurbita moschata* Duchesne ex Poir) according to the ripening process. 30 days after flowering the concentration of alpha-tocopherol amounted to 94.4 mg/100 g. The content of gamma-tocopherol was 94.47 mg/100 g, whereas the concentration of sigma-tocopherol was low, i.e., 5.02 mg/100 g. It can be observed that the concentration of tocopherols in pumpkin seeds and pumpkin seed oils is about 10–40 times higher than in pumpkin flesh (based on dry weight). The research showed that as the growth period became longer, the content of tocopherols decreased significantly. The concentrations of tocopherols measured by Procida et al. [49] and by Petkova and Antova [50] were higher than the contents of alpha- and gamma-tocopherols found in the pumpkin cultivars in our study.

### 2.5. Vitamins Content in C. pepo L. and C. moschata Duchesne ex Poir

The pumpkin cultivars were also analyzed for the content of vitamins C, B1 and folates (Table 4). All the cultivars had high content of vitamin C. The highest contents of ascorbic acid were observed in ‘Butternut’ and ‘Spaghetti’, whereas the lowest contents were found in ‘Orange Butternut’ and ‘Casperita’. All the pumpkins contained thiamine, but its concentration was relatively low and ranged from 0.15 to 0.72 mg/100 g in individual cultivars. The pumpkins were analyzed for the content of the following folates: folic acid, 5-methyltetrahydrofolate and tetrahydrofolate. Only 5-methyltetrahydrofolate was identified in the pumpkins. The highest contents of the compound were found in ‘Shishigatani’, ‘Festival’, and ‘Butternut’, whereas the lowest amounts were measured in ‘Spaghetti’, ‘Orangita’, and ‘Delicata’.

Studies on the content of vitamin C in various pumpkin cultivars from Ethiopia showed that their pulp contained low amounts of this vitamin (4.8–9.1 mg/100 g fresh weight) [51]. Blessing, Ifeanyi, and Chijioke [52] found small amounts of ascorbic acid in their study on pumpkins. The content ranged from 3.47 to 4.38 mg/100 g fresh weight. Ellong et al. [53] detected traces of vitamin C in the *Cucurbita moschata* pumpkin pulp (0.1 mg/100 g fresh weight). Echessa et al. [54] noted the presence of vitamin B1 in pumpkin pulp. The significant difference between the vitamin C content found in our studies, and the vitamin C content in other studies due to the fact that the results are presented in terms of dry or fresh mass. The authors indicated that the content of thiamine ranged from 0.285 to 0.880 mg/100 g, depending on the cultivar. According to data in reference publications, the content of folates in various pumpkin cultivars ranged from 12 to 27 μg/100 g (USDA). Based on our results and results from other authors, it can be unequivocally stated that pumpkin (even in dried form) is not a source of group B vitamins (thiamine and folate) regardless of its variety.

### 2.6. Minerals Content in C. pepo L. and C. Moschata Duchesne ex Poir

As far as minerals are concerned (Table 5), potassium was predominant. Its concentration ranged from 4104.3 to 7386.9 mg/100 g in different cultivars. The highest contents of potassium were found in ‘Butterkin’, ‘Casperita’, ‘Spaghetti’, and ‘Table Queen’. The lowest contents of this component were found in ‘Shishigatani’, ‘Futsu’, ‘Delicata’, ‘Festival’, and ‘Orange Butternut’. The highest contents of calcium were found in ‘Miranda’, ‘Spaghetti’, ‘Butterkin’, and ‘Baby Boo’. The lowest concentrations of this component were found in ‘Delicata’, ‘Orange Butternut’ and ‘Butternut’. ‘Miranda’, ‘Baby Boo’, and ‘Yuxijiangbinggua’ proved to be good sources of magnesium. The contents of magnesium were nearly two times lower in ‘Festival’, ‘Futsu’, ‘Butternut’, and ‘Table Queen’. ‘Baby Boo’ and ‘Butterkin’ had the highest contents of sodium. The lowest concentrations of this component were found in ‘Festival’, ‘Futsu’, and ‘Casperita’. The highest contents of iron were found in ‘Miranda’, ‘Yuxijiangbinggua’, and ‘Orange Butternut’. They were the lowest in ‘Casperita’ and ‘Table Queen’. The following cultivars were the richest in zinc: ‘Casperita’, ‘Orangita’, and ‘Spaghetti’. The highest contents of copper were found in ‘Snow Ball’, ‘Shishigatani’, and ‘Futsu’. There were also small amounts of manganese. The highest concentrations of this component were found in ‘Spaghetti’, ‘Snow Ball’, and ‘Festival’.

Blessing et al. observed that potassium was the predominant mineral component in pumpkin pulp (*Cucurbita spp*.) [52]. Its concentration ranged from 123.89 to 217.669 mg/100 g fresh weight. The content of calcium ranged from18.5–24.4 mg/100 g fresh weight. Apart from that, there was low content of sodium. Like pulp, pumpkin seeds proved to have high potassium content (5790 μg/g). The research showed that they were also a good source of magnesium (5690 µg/g). They contained smaller amounts of calcium (346 μg/g), zinc (113 μg/g), and iron (106 μg/g) [55]. El-Adawy and Taha [56] noted high potassium content (982 mg/100 g) in pumpkin seed flour. However, the content was lower than in the pumpkin cultivars in our study. The authors of the study also found high amounts of magnesium (483 mg/100 g), which was about 4–5 times greater than in the pumpkin pulp [56]. The authors of the study also found calcium (130 mg/100 g) and sodium (38 mg/100 g). Their contents were lower than in all the pumpkin cultivars. They also identified iron (10.9 mg/100 g), manganese (8.9 mg/100 g), zinc (8.2 mg/100 g), and copper (1.7 mg/100 g). The amounts of these components were several times greater than in the pumpkin pulp.

### 2.7. Comparison of the Analyzed Compounds Content between Two Pumpkin Species

The content of bioactive compounds in the two pumpkin species was compared (Table 6). The table demonstrated that cultivar exert great influence as compared to species. It also demonstrates a high dispersion of results and, thus, relevant variability in the different plants. There were statistically significant differences (*p* = 0.05) in the content of various components. The content of the following carotenoids in *Cucurbita pepo* species was higher than in *Cucurbita moschata*: zeaxanthin (56.69 vs. 26.44 µg/g), lutein (135.28 vs. 68.69 µg/g) and beta-carotene (46.83 vs. 29.19 µg/g). Apart from that, *Cucurbita pepo* L. had higher contents of caffeic acid (72.06 vs. 32.18 mg/100 g), quercetin (3.29 vs. 1.07 mg/100 g), calcium (252.89 vs. 197.17 mg/100 g), and zinc (1.03 vs. 0.96 mg/100 g). On the other hand, *Cucurbita pepo* L. contained more phenolic acids than *Cucurbita moschata Duchesne ex Poir*: protocatechuic acid (15.96 vs. 24.96 mg/100 g), p-coumaric acid (0.36 vs. 1.11 mg/100 g) and sinapic acid (6.32 vs. 13.84 mg/100 g).

The correlation analysis revealed numerous statistically significant connections between the contents of individual bioactive compounds with antioxidative properties. For the *Cucurbita pepo* L. cultivars there was a strong positive correlation between the concentration of iron and manganese (r = 0.61; *p* < 0.01) and between the concentration of tocopherols and zinc (r = 0.81; *p* < 0.01) (Table 7). There were also positive correlations between the content of carotenoids and phenolic acids (r = 0.44, *p* < 0.05), between the content of phenolic acids and vitamin C (r = 0.42, *p* < 0.05), and also between the content of copper and manganese (r = 0.38, *p* < 0.05). On the other hand, the zinc content was negatively correlated with the content of phenolic acids (r = −0.36; *p* < 0.05), manganese (r = −0.40; *p* < 0.05), and iron (r = −0.61, *p* < 0.01). Apart from that, the high content of carotenoids was correlated with the low content of manganese (r = −0.55; *p* < 0.01) and zinc (r = −0.47; *p* < 0.05). Tocopherols were negatively correlated with the content of iron (r = −0.48, *p* < 0.05). Different correlations were found for *Cucurbita moschata* Duchesne (Table 8). Strong correlation was observed between the content of carotenoids and flavonols (r = 0.91, *p* < 0.001). We have also found a positive association between the content of tocopherols and iron (r = 0.76; *p* < 0.01), and manganese (r = 0.71; *p* < 0.01). On the other hand, the tocopherols content was negatively correlated with the content of copper (r = −0.67; *p* < 0.01). We have also showed positive correlations between iron and phenolic acids level (r = 0.63; *p* < 0.01), and between iron and manganese level (r = 0.59; *p* < 0.01). In addition, we have showed a negative correlation between the content of carotenoids and manganese (r = −0.59; *p* < 0.01), iron and copper (r = −0.61; *p* < 0.01), and zinc and copper (r = −0.58; *p* < 0.05). These results confirm the significant differences in the content of selected bioactive compounds in two species of pumpkin. The observed positive and negative correlations between the contents of the tested compounds may result from their antioxidative and prooxidative effects.

### 2.8. Cluster Analysis Results

**Figure 1:**
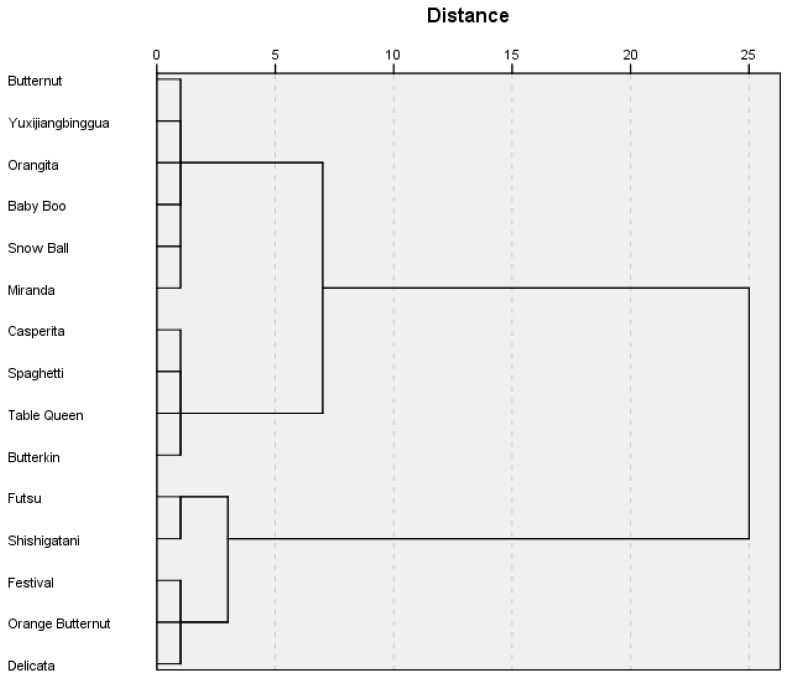
Dendrogram of studied pumpkin variety divisions.

A hierarchical cluster analysis was conducted according to Ward’s method in order to group the pumpkin cultivars according to the content of the bioactive components under analysis. The number and inclusion in groups was determined according to the dendrogram (Figure 1). Two groups of cultivars were clearly distinguished. The following cultivars were included in Cluster 1: ‘Baby Boo’, ‘Snow Ball’, ‘Miranda’, ‘Orangita’, ‘Butternut’, and ‘Yuxijiangbinggua’. The following cultivars were included in Cluster 2: ‘Butterkin’, ‘Table Queen’, ‘Casperita’, ‘Spaghetti’, ‘Shishigatani’, ‘Futsu’, ‘Delicata’, ‘Orange Butternut’, and ‘Festival’. Next, the clusters were analyzed for the variables which caused differences between the groups. The analysis showed statistically significant differences in the content of lutein, protocatechuic acid, alpha-tocopherol, alpha-tocopherol equivalent, myricetin, quercetin, potassium, and calcium (Table 9). The content of the following compounds in the first group was significantly higher than in the second group: lutein, alpha-tocopherol, alpha-tocopherol equivalent, myricetin, quercetin, potassium, and calcium. On the other hand, the second group was characterized by higher content of protocatechuic acid and folates.

## 3. Materials and Methods

### 3.1. Sample Collection and Preparation

The pulp of 15 cultivars of two pumpkin species *Cucurbita pepo* L. (Spaghetti, Miranda, Snow Ball, Orangita, Casperita, Baby Boo, Delicata, Table Queen, Festival) and *Cucurbita moschata* Duchesne ex Poir (Butternut, Butterkin, Futsu, Shishigatani, Orange Butternut, Yuxijiangbinggua) was used as the research material. All the pumpkin cultivars were purchased at the ‘Dolina Mogilnicy’ Organic Farming Products Cooperative (Wolkowo, Poland, 52°9′23.27″ N; 16°30′10.69″E). The climate of Wolkowo is as follows: an average annual temperature of 8.9 °C, an average relative humidity of 78.0%, wind velocity of 3.0 m/s, and total annual rainfall of 515 mm). Sowing seeds of all pumpkin varieties took place in the same month (May 2016). Three to four seeds were sown in the nests, with a spacing of approx. 1 × 1 m. For all varieties the growing conditions were similar. During cultivation the plants were irrigated, weeded and the soil was loosened. The pumpkins were harvested in October 2016. On harvesting they were transported to the university and cleaned. The experimental unit consisted of two randomly chosen pumpkins from each variety. All chemical analyses for each pumpkin were performed in triplicate. Pumpkins were stored in a cold room at 3–4 °C, without light exposure, and relative humidity of about 85%. The edible pulp was cut into pieces and lyophilized. The dried pulp was stored at room temperature without access to air and light. 

### 3.2. Reagents

Reagents (acetonitrile, triethylamine, sulfuric acid, Taka-Diastase, potassium ferricyanide, ethyl acetate, pyrogallol, ethanol, isobutanol, potassium hydroxide, orthophosphoric acid, methanol, nitric acid, lanthanum chloride, meta-phosphoric acid, potassium dihydrogen phosphate) were obtained from Sigma Aldrich (Poznań, Poland).

### 3.3. Determination of Water Content

The water content was determined by the drying method. For this purpose, 1 g ± 0.001 g of pumpkin pulp was weighed into a weighing bottle and mass was recorded. Then, the samples were heated to 105 °C and dried for three hours. After this time, weighing bottles with samples were transferred to a desiccator (cooling time was approx. 45–60 min). Once cooled, weighing bottles were weighed and the results were recorded. Then the weighing bottle was put back into the dryer and dried for 30 min. After this time the weighing bottles were again transferred to the desiccator and then weighed. The procedure was continued until the next two determinations differed less than 0.004 g. Each sample was measured in triplicate.

### 3.4. HPLC Analysis of Carotenoids

Two grams of samples and 0.5 g of pyrogallol were weighed into round bottom flasks. Then 20 mL of ethyl alcohol (anhydrous) and 2 mL of 60% KOH were added. The thus-prepared sample was heated for 30 min at the solvent boiling point. Then 50 mL of 1% NaCl was added to the samples and cooled. Then 50 mL of n-hexane with 10% ethyl acetate was added. The closed flasks were shaken (300 rpm) for 30 min. Then 2 mL of saturated NaCl solution was added. After saponification 20 mL was withdrawn from the top layer into a round bottom flask to evaporate the solvent in a vacuum evaporator. The sample was then quantitatively transferred with 2 mL of ethyl acetate to the chromatographic vials. The content of carotenoids was measured by means of high-performance liquid chromatography (HPLC) with a PDA 2998 detector (Waters, Milford, MA, USA), Waters 600 pump, Waters 2707 autosampler and an RP-18 ODS2 column (250 × 4.6 mm; 5 µm) (Waters, Milford, MA, USA) working in 25 °C. Solvent A – 80% acetonitrile with 0.05% triethylamine and solvent B – ethyl acetate were used in the elution process. The following gradients were used: 65% A: 35% B for 35 minutes, 50% A: 50% B for the next 25 min. Then this proportion was maintained in the isocratic system for another 5 min at a flow rate of 1.0 mL/min. The registration wavelength was λ = 450 nm. Compounds were identified according to their spectra ranging from 200 to 600 nm and retention times compared with standards [57]. As an internal standard, β-carotene, lutein and zeaxanthin were used (SigmaAldrich Chemie GmbH, Steinheim, Germany). Based on the carotenoids content (beta-carotene, lutein, zeaxanthin) the equivalent of retinol was calculated. According to literature data, it is assumed that 1 μg retinol equivalent (RE) = 6 μg beta-carotene + 12 μg other carotenoids [58].

### 3.5. HPLC Analysis of Phenolic Acids

To determine the phenolic compounds content, 1 g of sample was weighed and extracted with 32 mL of 80:20 methanol/water (*v*/*v*) using an ultrasonic water bath. Extraction was carried out at room temperature for 30 minutes. The extract was centrifuged (4000 rpm/10 min). The solvent was removed under vacuum. The residue was dissolved in 1 mL of 80:20 methanol:water (*v*/*v*). The sample was filtered using a syringe filter (PTFE).

The content of phenolic acids was measured using HPLC by means of a liquid chromatograph (Agilent) with an Infinity Bin Pump DAD 1290 detector (Agilent Technology, Santa Clara, CA, USA) and a Zorbax SB C18 column (3.9 × 150 mm, 5 μm) (Agilent Technology, Santa Clara, CA, USA) working in 22 °C. The following non-linear concentration gradient was used: water acidified with orthophosphoric acid (0.05%) to pH 2.7 and acetonitrile with water (1:1 *v*/*v*) at a flow rate of 1.5 mL/min. The gradient program started with 100% of acidified water and ended with 50% of acetonitrile at the 52nd minute of the separation. Compounds were identified by comparing their retention times with retention times of the standards (chlorogenic acid (5-O-dicuaoylquinic acid), ferulic acid (4-hydroxy-3-methoxycinnamic acid), gallic acid (3,4,5-trihydroxybenzoic acid), caffeic acid (3,4-hydroxycinnamic acid), p-hydroxybenzoic acid (4-hydroxybenzoic acid), p-coumaric acid (4-hydroxycinnamic acid), protocatechic acid (3,4-dihydroxybenzoic acid), sinapic acid (4-hydroxy-3,5-dimethoxycinnamic acid), vanillic acid (4-hydroxy-3-methoxybenzoic acid)) (Sigma-Aldrich, Poznań, Poland) dissolved in methanol. The identification of individual phenolic acids consisted in comparing the UV–VIS spectrum and the retention time of acids in the samples with the UV–VIS spectrum and the retention time of standards measured at wavelengths of 260 and 310 nm [59]. The concentration of phenolic acids in samples were calculated using an external standard.

### 3.6. HPLC Analysis of Flavonols

The content of flavonols was determined according to the same conditions as phenolic acids. The content of flavonols was measured at a flow rate of 1.0 mL/min. The gradient programme started at the 1st minute with 95% of acidified water and it decreased to 20% at the 30th minute. The compounds were detected at a wavelength of 370 nm [60]. Compounds were identified by comparing their retention times with retention times of the standards (astragalin (kempferol 3-O-glucopyranoside), isoquercetin (quercetin-3-O-glucosidey), kaempferol (3,4′,5,7-tetrahydroxyflavone), quercetin (3,3′4′,5,7-pentahydroxyflavone), rutin (quercetin 3-rutinoside), myrycetin) (Sigma-Aldrich, Poznań, Poland) dissolved in methanol. The concentration of flavonols in samples were calculated using an external standard.

### 3.7. HPLC Analysis of Tocopherols

Two grams of samples and 0.5 g of pyrogallol were weighed into round bottom flasks. Then 20 mL of ethyl alcohol (anhydrous) and 2 mL of 60% KOH were added. Thus prepared sample was heated for 30 min at the solvent boiling point. Then 50 mL of 1% NaCl was added to the samples and cooled. Then 50 mL of n-hexane with 10% ethyl acetate was added. The closed flasks were shaken (300 rpm) for 30 min. Then 2 mL of saturated NaCl solution was added. After saponification 20 mL was withdrawn from the top layer into a round bottom flask to evaporate the solvent in a vacuum evaporator. Tocopherols were extracted and identified following the procedure described by Ryynane et al. [60] with modifications. Tocopherols were identified by means of a high performance liquid chromatograph (Waters 600 Asc. Milford, MA, USA) with a Waters 600 pump, LiChrosorb Si 60 column (200 × 4.6 mm, 5 μm, Merck, Darmstadt, Germany) and a fluorometric detector (Waters 474 Asc. Milford, MA, USA). A mixture of n-hexane with 1,4-dioxane (96:4 *v*/*v*) was the mobile phase. The flow rate was 1.0 mL/min. The detector worked at an excitation λ = 290 nm and emission λ = 330 nm. The concentration of individual tocopherol homologues was calculated by means of a calibration curve [60]. The following standards were used to evaluate tocopherols content: α-, β-, γ-, and δ-tocopherol (>95% purity) (Merck, Darmstadt, Germany). Based on the tocopherols content, the equivalent of alpha-tocopherol was calculated. According to literature (FAO, Food and Agriculture Organization of the United Nations), it is assumed that 1 mg alpha-tocopherol equivalent (α-TE) = 1 mg α-tocopherol + 0.5 mg β-tocopherol + 0.1 mg γ -tocopherol [61].

### 3.8. Mineral Composition

The content of mineral components (Ca, Mg, Mn, Fe, Zn, Cu) was measured by means of flame atomic absorption spectrometry (F-AAS), whereas the contents of Na and K were measured by means of flame atomic emission spectrometry (F-AES). An AAS-3 spectrophotometer (Carl-Zeiss Jena, Germany) was used for the analysis. The samples had undergone wet mineralization with spectrally-pure, concentrated 65% nitric acid in a closed system of a MARS 5 microwave furnace (CEM, Matthews, NC, USA). Mineralization was carried out at 210 °C for 10 min (1200 W) [46].

### 3.9. HPLC Analysis of Vitamin C

To determine the vitamin C content, 0.5 g of the sample was weighed and extracted with 2 mL of 1% metaphosphoric acid using ultrasonic water bath (15 min). Then 1 mL of metaphosphoric acid was added to the sample and it was placed in the bath again. After 15 minutes, 5 mL of metaphosphoric acid was added. Then, the sample was centrifuged. Thero1,4-dimercapto-2,3-butandiol (0.2 mL) was added to the supernatant (0.2 mL) and then diluted with water to 2 mL. The content of vitamin C was measured with a high-performance liquid chromatograph (Agilent Technology, Santa Clara, CA, USA) with an Infinity Bin Pump DAD 1290 detector and a Luna Phenomenex column (4.6 × 250 mm, 5 μm) (Torrance, CA, USA). A mixture of potassium dihydrogen phosphate (pH = 5.0) and methanol was the mobile phase (0.05 mol/L KH_2_PO_4_:methanol, 97:3). The gradient of the potassium dihydrogenphosphate mixture ranged from 95% at the first min. up to 78% at the sixth min. The content of ascorbic acid was measured at a wavelength of 245 nm using the previously prepared standard curve [62].

### 3.10. Spectrofluorometric Measurement of Thiamine (Vitamin B1)

To determine thiamine content, the pumpkin samples were extracted by two-step hydrolysis. Acid hydrolysis was carried out using 0.2M H_2_SO_4_, heating the sample at 121 °C for 30 min. Then enzymatic hydrolysis was performed using Taka Diastase (10% solution) at pH 4.5 using 2.5M CH_3_COONa. Potassium ferricyanide was added to the filtrate in order to oxidize thiamine to the thiochrome. The thiochrome were then extracted with isobutanol. The mixture was allowed to separate the layers. Then 1 mL (upper layer) was collected and the fluorescence was measured. The content of thiamine was measured with the thiochrome method, which included the analysis of quantitative changes in the free and bound form. The samples were measured fluorometrically at an excitation wavelength λ = 365 nm and by means of a secondary filter with the maximum permeability at a wavelength λ = 435 nm [63].

### 3.11. HPLC analysis of Folates

Folic acid, 5-methyltetrahydrofolate, and tetrahydrofolate were prepared according to the method described by Konings [64]. The concentration of standards was calculated with molar absorption coefficients [65]. Alpha-amylase (E.C. 3.2.1.1, A-6211) was dissolved in 0.1 M phosphate buffer (pH = 7.0) immediately before analysis. Gamma-glutamyl hydrolase was acquired from rat blood plasma (Europa Bioproducts Ltd., Cambridge). Samples were prepared in a darkened room. About 0.5 g of the sample was weighed into test tubes. Next, 0.1 M phosphate buffer with 2% (*w*/*v*) ascorbic acid and 0.2% (*v*/*v*) 2-mercaptoethanol was added. The samples were shaken and simultaneously they were heated in a water bath at 100 °C for 15 minutes. After that, the samples were cooled to 20 °C and gamma-glutamyl hydrolase and alpha-amylase were added. The samples were incubated at 37 °C for four hours. Next, they were heated for five minutes at 100 °C and cooled down. After that, the samples were centrifuged for 20 min (12,000 rpm) at 4 °C. The supernatant was poured into dark glass measuring flasks. A total of 0.1 M phosphate buffer was added to the residue left in the test tubes. The samples were shaken and centrifuged again. The supernatant was poured into measuring flasks again and filtered [66]. The samples were purified on Bakerbond spe J. T. columns (Radnor, PA, USA). The folates were separated with a Shimadzu LC-10A liquid chromatograph (Nakagyo-ku, Kyoto, Japan) with a Phenomenex Synergi 4a Hydro-RP 80A column (4 μm, 250 × 4.6 mm; Torrance, CA, USA). The separation was conducted according to the procedure described by Jastrebowa et al. [67]. The folates were identified and their content was measured according to the standard with a known content of folates.

### 3.12. Limits of Detection (LOD) and Limits of Quantification (LOQ)

Limits of detection (LOD) and limits of quantification (LOQ) were calculated according to Seal [68].

### 3.13. Statistical Analysis

The results were analyzed statistically by means of the STATISTICA 13.1 program (StatSfot, Inc., Kraków, Poland). The experimental unit consisted of two randomly chosen pumpkins from each variety. All chemical analyses for each pumpkin were made in triplicate. Analysis of variance was conducted to detect statistically significant differences between the varieties. The multiple comparison analysis was conducted with post-hoc LSD tests. The significance level was assumed at *p* = 0.05. Pearson’s linear correlation coefficients (*p* = 0.05, *p* = 0.01, *p* = 0.001) between the contents of individual compounds were calculated. A hierarchical cluster analysis was conducted according to Ward’s method. It let us group the pumpkin varieties according to the content of the components under analysis.

## 4. Conclusions

In this study, the profile of bioactive compounds in pumpkin flesh was characterized for the first time in such a wide range, comparing many varieties of pumpkins belonging to two species (*Cucurbita pepo* and *Cucurbita moschata*), which is a novelty in food analysis. The content of the following components was measured: carotenoids, flavonols, phenolic acids, tocopherols, mineral components, and vitamins. In most cases high-performance liquid chromatography was applied to measure the content of individual compounds. Apart from that, the content of compounds in the fifteen pumpkin cultivars was compared. Additionally, the profile of bioactive compounds in the *Cucurbita pepo* L. and *Cucurbita moschata* Duchesne ex Poir pumpkin species was compared. The research proved that pumpkin pulp is the source of many valuable components with documented health-beneficial properties. Due to the presence of many compounds with a well-documented antioxidant properties, pumpkin flesh can be a valuable component of the diet, involved in neutralizing free radicals, which contribute to the development of non-communicable diseases. The study also showed that the species and cultivars were characterized by high diversity in the content of compounds under analysis. As a result, it can be concluded that the selection of a specific pumpkin variety may be particularly important for human health in terms of the amount of bioactive compounds provided. Thus far there has not been such a detailed analysis of the content of bioactive compounds in pumpkin pulp, which are decisive to its health-promoting potential. Additionally, the profile of bioactive compounds has never been compared in so many pumpkin cultivars before. The results of the study are an element that broadens the current state of knowledge and indicate new research trends. It is worth undertaking the development of new food products containing pumpkin flesh (e.g., in powdered form), as an example of a functional food with specific, enhanced health properties. Thus far, not many food products based on pumpkin flesh have been developed. Few food products appearing in the literature were mainly examined in terms of their physicochemical properties. It seems that the subject of new food products based on pumpkin flesh has not been fully examined, especially in the context of the analysis of bioactive compounds. Although pumpkin is one of the well-known vegetables in the world, still not all aspects related to its health potential have been tested.

## Figures and Tables

**Table 1:** The content of carotenoids and moisture content in pumpkin varieties.

	Cultivars	Carotenoids (mg/100g dm)				Moisture Content (%)
		Zeaxanthin	Lutein	β-carotene	Retinol Equivalent	
Cucurbita pepo	Spaghetti	0.61 ± 0.01^a^	14.75 ± 0.06^a^	4.65 ± 0.13^a^	2.05 ± 0.02^a^	92.02 ± 0.80^a^
	Miranda	0.86 ± 0.01^b^	12.77 ± 0.06^b^	5.12 ± 0.04^b^	1.99 ± 0.01^b^	95.16 ± 0.76^b^
	Snow Ball	1.84 ± 0.00^c^	9.71 ± 0.03^c^	3.32 ± 0.04^c^	1.52 ± 0.01^c^	93.79 ± 0.71^c^
	Orangita	11.87 ± 0.04^d^	22.50 ± 0.03^d^	4.81 ± 0.10^d^	3.67 ± 0.01^d^	87.28 ± 0.78^d^
	Casperita	9.69 ± 0.04^e^	7.34 ± 0.05^e^	1.58 ± 0.05^e^	1.68 ± 0.01^e^	87.05 ± 0.85^e^
	Baby Boo	6.08 ± 0.13^f^	9.61 ± 0.04^c^	7.62 ±0.04^f^	2.58 ± 0.02^f^	83.70 ± 1.01^f^
	Delicata	5.03 ± 0.05^g^	13.28 ± 0.12^f^	5.33 ± 0.06^g^	2.41 ± 0.02^g^	82.35 ± 0.55^g^
	Table Queen	3.17 ± 0.03^h^	22.92 ± 0.08^g^	8.33 ± 0.07^h^	3.56 ± 0.02^h^	88.66 ± 0.62^h^
	Festival	12.12 ± 0.18^i^	8.86 ± 0.08^h^	1.39 ± 0.05^i^	1.98 ± 0.02^b^	87.33 ± 0.46^i^
Cucurbita moschata	Butternut	0.31± 0.00^j^	11.13 ± 0.08^i^	3.77 ± 0.07^j^	2.12 ± 0.02^i^	87.14 ± 0.70^j^
	Butterkin	0.31 ± 0.00^j^	3.34 ± 0.04^j^	1.29 ± 0.04^i^	0.52 ± 0.01^j^	87.99 ± 0.83^k^
	Futsu	3.87 ± 0.02^k^	6.34 ± 0.09^k^	3.56 ± 0.06^k^	1.44 ± 0.02^k^	82.52 ± 0.65^l^
	Shishigatani	0.39 ± 0.00^j^	4.24 ± 0.04^l^	1.72 ± 0.04^l^	0.67 ± 0.01^l^	84.49 ± 1.12^m^
	Orange Butternut	2.94 ± 0.03^l^	4.59 ± 0.02^m^	5.26 ± 0.08^g^	1.50 ± 0.02^c^	86.71 ± 0.47^n^
	Yuxijiangbinggua	1.52 ± 0.01^m^	11.56 ± 0.03^n^	1.91 ± 0.04^m^	1.41 ± 0.01^m^	91.52 ± 1.04^o^

a–o: means in the same column followed by the same letters shown in superscript do not significantly differ (*p* > 0.05) in terms of analyzed variables; dm: dry mass; the results are expressed as the mean values ± standard deviation of the triplicate samples.

**Table 2:** The content of phenolic acids in pumpkin varieties.

	Cultivar	Phenolic Acids (mg/100 g dm)								
		Gallic Acid	Protocatechuic Acid	4-Hydroxybenzoic Acid	Vanilic Acid	Chlorogenic Acid	Caffeic Acid	P-coumaric Acid	Ferulic Acid	Sinapic Acid
Cucurbita pepo	Spaghetti	7.72 ± 0.03^a^	27.49 ± 0.18^a^	17.00 ± 0.34^a^	1.39 ± 0^a^	4.04 ± 0.03^a^	98.86 ± 0.53^a^	0.04 ± 0.0^a^	5.09 ± 0.04^a^	11.12 ± 0.07^a^
	Miranda	6.50 ± 0.04^b^	11.1 ± 0.05^b^	4.03 ± 0.16^b^	3.06 ± 0.03^b^	8.00 ± 0.03^b^	101.82 ± 0.34^b^	0.94 ± 0.04^b^	41.41 ± 0.46^b^	n.d.
	Snow Ball	14.50 ± 0.04^c^	4.91 ± 0.03^c^	16.71 ± 0.25^a^	7.10 ± 0.05^c^	4.05 ± 0.04^a^	28.97 ± 0.33^c^	n.d.	1.90 ± 0.03^c^	n.d.
	Orangita	9.38 ± 0.03^d^	8.33 ± 0.06^d^	9.84 ± 0.05^c^	5.67 ± 0.03^d^	6.01 ±0.02^c^	118.83 ± 0.58^d^	0.92 ± 0.01^c^	6.17 ± 0.06^d^	n.d.
	Casperita	21.91 ± 0.04^e^	6.86 ± 0.06^e^	5.90 ± 0.14^d^	4.83 ± 0.06^e^	7.88 ± 0.04^d^	49.56 ± 0.48^e^	n.d.	6.01 ± 0.08^d^	7.24 ± 0.14^b^
	Baby Boo	11.18 ± 0.09^f^	19.00 ± 0.25^f^	5.54 ± 0.15^d^	1.07 ± 0.01^f^	3.52 ± 0.03^e^	75.06 ± 0.29^f^	0.16 ± 0.01^d^	9.09 ± 0.02^e^	n.d.
	Delicata	14.66 ± 0.04g	38.46 ± 0.45^g^	9.49 ± 0.13^c^	5.32 ± 0.01^g^	5.38 ± 0.01^f^	50.19 ± 0.13^g^	n.d.	12.39 ± 0.07^f^	11.32 ± 0.13^a^
	Table Queen	16.37 ± 0.04^h^	23.02 ± 0.31^h^	22.21 ± 0.15^e^	3.99 ± 0.01^h^	5.25 ± 0.02^g^	34.46 ± 0.34^h^	n.d.	40.90 ± 0.39^g^	27.24 ± 0.23^c^
	Festival	4.83 ± 0.05^i^	4.50 ± 0.11^i^	14.90 ± 0.42^f^	5.34 ± 0.02^g^	6.30 ± 0.06^h^	90.79 ± 0.57^i^	1.15 ± 0.02^e^	26.45 ± 0.10^h^	n.d.
Cucurbita moschata	Butternut	11.49 ± 0.15^j^	9.90 ± 0.01^j^	22.56 ± 0.56^e^	4.38 ± 0.02^i^	5.11 ± 0.06	16.75 ± 0.21^j^	3.06 ± 0.01^f^	26.86 ± 0.17^i^	27.58 ± 0.40^d^
	Butterkin	5.34 ± 0.02^k^	8.92 ± 0.02^k^	7.72 ± 0.07^g^	4.08 ± 0.03^j^	2.20 ± 0.01	11.79 ± 0.15^k^	n.d.	4.53 ± 0.06^j^	17.95 ± 0.11^e^
	Futsu	12.33 ± 0.12^l^	16.81 ± 0.02^l^	11.32 ± 0.14^h^	5.35 ± 0.03^g^	3.56 ± 0.03^e^	28.01 ± 0.12^l^	n.d.	12.53 ± 0.17^f^	n.d.
	Shishigatani	25.62 ± 0.06^m^	52.55 ± 0.04^m^	9.87 ± 0.04^c^	9.49 ± 0.02^k^	7.10 ± 0.02	8.96 ± 0.07^m^	1.90 ± 0.01^g^	n.d.	n.d.
	Orange Butternut	8.92 ± 0.03^n^	33.63 ± 0.42^n^	14.12 ± 0.50^i^	0.48 ± 0.02^l^	7.85 ± 0.01^d^	53.04 ± 0.39^n^	0.66 ± 0.01^h^	22.45 ± 0.04^k^	23.14 ± 0.19^f^
	Yuxijiangbinggua	5.31 ± 0.01^k^	27.93 ± 0.06^o^	28.99 ± 0.22^j^	3.96 ± 0.01^h^	4.02 ± 0.01^a^	74.52 ± 0.42^f^	1.03 ± 0.01^i^	9.02 ± 0.02^e^	14.39 ± 0.17^g^

a–n: means in the same column followed by the same letters shown in superscript do not significantly differ (*p* > 0.05) in terms of analyzed variables; dm: dry mass; n.d.—not detected; the results are expressed as the mean values ± standard deviation of the triplicate samples.

**Table 3:** The content of flavonols in pumpkin varieties.

	Cultivars	Flavonols (mg/100 g dm)					
		Rutin	Kaempferol	Isoquercetin	Astragalin	Myricetin	Quercetin
Cucurbita pepo	Spaghetti	7.96 ± 0.03^a^	4.96 ± 0.04^a^	n.d.	4.12 ± 0.03^a^	n.d.	n.d.
	Miranda	2.02 ± 0.04^b^	n.d.	n.d.	n.d.	3.12 ± 0.06^a^	n.d.
	Snow Ball	31.91 ± 0.04^c^	n.d.	3.90 ± 0.02^a^	10.04 ± 0.09^b^	n.d.	9.81 ± 0.03^a^
	Orangita	14.17 ± 0.04^d^	16.98 ± 0.04^b^	3.45 ± 0.01^b^	19.40 ± 0.02^c^	4.82 ± 0.03^b^	n.d.
	Casperita	17.29 ± 0.06^e^	n.d.	3.94 ± 0.02^a^	n.d.	n.d.	6.29 ± 0.02^b^
	Baby Boo	13.33 ± 0.04^f^	n.d.	n.d.	6.33 ± 0.09^d^	8.85 ± 0.03^c^	5.66 ± 0.04^c^
	Delicata	8.97 ± 0.06^g^	n.d.	4.91 ± 0.03^c^	n.d.	3.43 ± 0.02^d^	n.d.
	Table Queen	5.38 ± 0.02^h^	29.90 ± 0.05^c^	n.d.	3.00 ± 0.04^e^	4.29 ± 0.04^e^	3.33 ± 0.01^d^
	Festival	13.13 ± 0.11^i^	36.24 ± 0.08^d^	2.36 ± 0.05^d^	2.68 ± 0.01^f^	n.d.	4.51 ± 0.02^e^
Cucurbita moschata	Butternut	46.93 ± 0.13^j^	18.91 ± 0.01^e^	1.00 ±0.01^e^	n.d.	n.d.	4.51 ± 0.03^e^
	Butterkin	5.4 ± 0.01^h^	1.06 ± 0.01^f^	n.d.	6.65 ± 0.04^g^	3.34 ± 0.05^f^	n.d.
	Futsu	15.32 ± 0.07^k^	n.d.	n.d.	16.31 ± 0.03^h^	0.89 ± 0.01^g^	n.d.
	Shishigatani	3.51 ± 0.01^l^	n.d.	n.d.	n.d.	n.d.	n.d.
	Orange Butternut	18.22 ± 0.07^m^	22.68 ± 0.04^g^	4.95 ± 0.03^c^	22.42 ± 0.02^i^	n.d.	n.d.
	Yuxijiangbinggua	3.99 ± 0.02^n^	14.00 ± 0.04^h^	n.d.	n.d.	4.09 ± 0.04^h^	1.92 ± 0.02^f^

a–n: means in the same column followed by the same letters shown in superscript do not significantly differ (*p* > 0.05) in terms of analyzed variables; dm: dry mass; n.d.: not detected; the results are expressed as the mean values ± standard deviation of the triplicate samples.

**Table 4:** The content of tocopherols, vitamin C, B1, and folates in pumpkin varieties.

	Cultivars	Tocopherols (mg/100 g dm)			Vitamin C (mg/100 g dm)	Vitamin B1 (mg/100 g dm)	Folates (ug/100 g dm)
		α-tocopherol	γ-tocopherol	α-tocopherol equivalent			
Cucurbita pepo	Spaghetti	6.44 ± 0.02^a^	8.07 ± 0.06^a^	7.25 ± 0.03^a^	82.89 ± 0.79^a^	0.72 ± 0.03^a^	18.89 ± 0.21^a^
	Miranda	2.58 ± 0.03^b^	0.68 ± 0.04^b^	2.65 ± 0.02^b^	68.05 ± 0.28^b^	0.15 ± 0.02^b^	44.58 ± 1.02^b^
	Snow Ball	1.69 ± 0.01^c^	4.23 ± 0.03^c^	2.11 ± 0.01^c^	65.56 ± 0.68^c^	0.33 ± 0.02^c^	32.22 ± 0.81^c^
	Orangita	3.92 ± 0.03^d^	2.49 ± 0.02^d^	4.17 ± 0.03^d^	66.71 ± 0.41^bc^	0.26 ± 0.01^d^	21.84 ± 0.60^d^
	Casperita	6.42 ± 0.03^a^	3.56 ± 0.02^e^	6.78 ± 0.04^e^	51.31 ± 1.21^d^	0.39 ± 0.02^e^	45.93 ± 0.35^e^
	Baby Boo	5.29 ± 0.01^e^	0.96 ± 0.01^f^	5.38 ± 0.01^f^	56.02 ± 0.67^e^	0.33 ± 0.01^c^	29.40 ± 0.47^f^
	Delicata	3.87 ± 0.02^f^	5.34 ± 0.02^g^	4.40 ± 0.02^g^	70.32 ± 0.90^f^	0.15 ± 0.01^b^	22.71 ± 0.39^d^
	Table Queen	2.96 ± 0.01^g^	2.68 ± 0.03^h^	3.23 ± 0.01^h^	55.78 ± 0.69^e^	0.24 ± 0.01^f^	35.97 ± 0.33^g^
	Festival	1.71 ± 0.01^cj^	1.50 ± 0.02^i^	1.86 ± 0.01^i^	67.93 ± 0.48^bc^	0.56 ± 0.02^g^	59.16 ± 0.26^h^
Cucurbita moschata	Butternut	3.08 ± 0.05^h^	0.65 ± 0.01^b^	3.14 ± 0.05^j^	83.05 ± 0.59^a^	0.33 ± 0.01^c^	58.78 ± 0.74^h^
	Butterkin	2.59 ± 0.02^b^	6.34 ± 0.04^j^	3.23 ± 0.01^h^	73.13 ± 0.98^g^	0.46 ± 0.02^h^	43.22 ± 0.36^i^
	Futsu	1.93 ± 0.02^i^	2.39 ± 0.04^k^	2.17 ± 0.02^k^	66.63 ± 0.^47bc^	0.20 ±0.01^i^	56.22 ± 0.71^j^
	Shishigatani	1.73 ± 0.01^j^	0.71 ± 0.01^b^	1.80 ± 0.01^l^	61.18 ± 1.37^h^	0.29 ± 0.01^j^	60.61 ± 0.74^k^
	Orange Butternut	4.38 ± 0.02^k^	9.96 ± 0.09^l^	5.38 ± 0.03^f^	41.98 ± 1.26^i^	0.62 ± 0.02^k^	49.68 ± 0.57^l^
	Yuxijiangbinggua	5.85 ± 0.01^l^	7.46 ± 0.02^m^	6.60 ± 0.01^m^	73.91 ± 0.46^g^	0.33 ± 0.01^c^	25.79 ± 0.22^m^

a–m: means in the same column followed by the same letters shown in superscript do not significantly differ (*p* > 0.05) in terms of analyzed variables; dm: dry mass; the results are expressed as the mean values ± standard deviation of the triplicate samples. δ-tocopherol and β-tocopherol were not detected in the evaluated samples.

**Table 5:** The content of mineral compounds in pumpkin varieties.

	Cultivar	Mineral compound (mg/100 g dm)							
		K	Ca	Mg	Na	Fe	Zn	Cu	Mn
Cucurbita pepo	Spaghetti	7118.37 ± 26.22^a^	331.99 ± 3.05^a^	128.27 ± 1.57^a^	261.88 ± 0.54^a^	1.80 ± 0.02^a^	1.19 ± 0.01^a^	0.35 ± 0.01^a^	0.85 ± 0.02^a^
	Miranda	5958.97 ± 10.21^b^	503.56 ± 2.51^b^	151.25 ± 0.88^b^	280.75 ± 1.41^b^	2.59 ± 0.03^b^	0.54 ± 0.02^b^	0.28 ± 0.01^b^	0.55 ± 0.01^b^
	Snow Ball	5811.47 ± 18.10^c^	208.59 ± 2.94^c^	104.78 ± 0.80^c^	297.44 ± 0.92^c^	2.42 ± 0.01^c^	0.95 ± 0.02^c^	0.63 ± 0.01^c^	0.70 ± 0.01^c^
	Orangita	6263.37 ± 22.60^d^	240.65 ± 2.59^d^	87.57 ± 1.72^d^	260.39 ± 1.04^a^	1.27 ± 0.01^d^	1.24 ± 0.01^d^	0.28 ± 0.02^b^	0.28 ± 0.01^d^
	Casperita	7229.07 ± 22.33^e^	201.37 ± 1.39^e^	116.29 ± 0.55^e^	220.08 ± 0.83^d^	1.04 ± 0.03^e^	1.31 ± 0.04^e^	0.36 ± 0.01^d^	0.37 ± 0.01^e^
	Baby Boo	5944.40 ± 13.69^b^	279.91 ± 1.80^f^	147.75 ± 1.18^f^	347.81 ± 0.85^e^	2.37 ± 0.02^f^	1.11 ± 0.01^f^	0.21 ± 0.01^e^	0.53 ± 0.02^f^
	Delicata	4883.47 ± 15.90^f^	133.29 ± 4.10^g^	131.72 ± 0.61^g^	319.24 ± 0.39^f^	1.48 ± 0.01^g^	1.17 ± 0.02^g^	0.22 ± 0.01^f^	0.34 ± 0.01^g^
	Table Queen	7004.93 ± 12.61^g^	173.43 ± 3.41^h^	83.79 ± 0.63^h^	329.53 ± 1.98^g^	1.23 ± 0.02^h^	0.81 ± 0.02^h^	0.39 ± 0.01^g^	0.47 ± 0.02^h^
	Festival	5237.80 ± 32.84^h^	203.24 ± 1.95^e^	80.78 ± 1.71^h^	217.36 ± 1.34^h^	2.35 ± 0.01^i^	0.96 ± 0.02^c^	0.29 ± 0.01^h^	0.69 ± 0.02^c^
Cucurbita moschata	Butternut	6445.63 ± 12.17^i^	155.71 ± 3.50^i^	82.49 ± 1.55^h^	284.74 ± 1.44^i^	1.80 ± 0.01^a^	1.04 ± 0.01^i^	0.35 ± 0.01^ad^	0.30 ± 0.01^i^
	Butterkin	7386.90 ± 20.24^j^	281.23 ± 2.29^f^	99.12 ± 1.08^i^	346.92 ± 0.38^e^	1.83 ± 0.03^j^	1.16 ± 0.01^g^	0.28 ± 0.01^b^	0.59 ± 0.01^j^
	Futsu	4371.20 ± 13.41^k^	244.18 ± 3.05^d^	81.15 ± 1.96^h^	219.05 ± 0.64^d^	1.24 ± 0.01^h^	0.87 ± 0.03^j^	0.53 ± 0.02^i^	0.38 ± 0.01^e^
	Shishigatani	4104.30 ± 11.94^l^	179.47 ± 2.56^j^	129.32 ± 0.89^a^	315.18 ± 0.13^j^	1.72 ± 0.02^k^	0.88 ± 0.01^j^	0.53 ± 0.02^i^	0.33 ± 0.01^g^
	Orange Butternut	5343.77 ± 18.21^m^	152.34 ± 2.52^i^	104.39 ± 1.05^c^	297.96 ± 1.35^c^	2.46 ± 0.01^l^	0.95 ± 0.02^c^	0.26 ± 0.01^j^	0.48 ± 0.02^h^
	Yuxijiangbinggua	6463.43 ± 12.47^i^	170.09 ± 3.82^h^	135.54 ± 1.56^j^	315.71 ± 0.52^j^	2.59 ± 0.03^b^	0.85 ± 0.02^k^	0.39 ± 0.01^g^	0.55 ± 0.01^b^

a–m: means in the same column followed by the same letters shown in superscript do not significantly differ (*p* > 0.05) in terms of analyzed variables; K: potassium, Ca: calcium, Mg- magnesium, Na: sodium, Fe: iron, Zn: zinc, Cu: copper, Mn: manganese; dm: dry mass; the results are expressed as the mean values ± standard deviation of the triplicate samples.

**Table 6:** Comparison of the bioactive compounds content between two pumpkin species.

	Compound	Cucurbita Pepo	Cucurbita Moschata	*P* Value
Carotenoids	Zeaxanthin	5.69 ± 4.38	2.64 ± 2.33	*p* < 0.05
	Lutein	13.53 ± 5.49	6.87 ± 3.38	*p* < 0.001
	β-carotene	4.68 ± 2.27	2.92 ± 1.44	*p* < 0.01
	Retinol equivalent	2.38 ± 0.74	1.28 ± 0.56	*p* < 0.05
Phenolic acids	Gallic acid	11.90 ± 5.24	11.50 ± 7.07	NS.
	Protocatechuic acid	15.96 ± 11.35	24.96 ± 15.7	*p* < 0.05
	4-Hydroxy-benzoic acid	11.74 ± 6.01	15.76 ± 7.79	NS.
	Vanillic acid	4.20 ± 1.94	4.62 ± 2.73	NS.
	Chlorogenic acid	5.60 ± 1.55	4.97 ± 2.03	NS.
	Caffeic acid	72.06 ± 31.15	32.18 ± 24.63	*p* < 0.001
	P-coumaric acid	0.36 ± 0.47	1.11 ± 1.12	*p* < 0.01
	Ferulic acid	16.60 ± 14.99	13.57 ± 8.57	NS.
	Sinapic acid	6.32 ±8.90	13.84 ± 10.92	*p* < 0.05
Tocopherols	α-tocopherol	3.88 ± 1.77	3.26 ± 1.49	NS.
	γ-tocopherol	3.28 ± 2.27	4.58 ± 3.65	NS.
	δ-tocopherol	n.d.	n.d.	-
	β-tocopherol	n.d.	n.d.	-
	α-tocopherol equivalent	4.20 ± 1.88	3.72 ± 1.77	NS.
Flavonols	Rutin	12.69 ± 8.30	15.56 ± 15.57	NS.
	Kaempferol	9.79 ± 13.83	9.44 ± 9.71	NS.
	Isoquercetin	2.06 ± 1.98	0.99 ± 1.86	NS.
	Astragalin	5.06 ± 6.07	7.56 ± 9.10	NS.
	Myricetin	2.72 ± 2.94	1.39 ± 1.74	NS.
	Quercetin	3.29 ± 3.43	1.07 ± 1.74	*p* < 0.05
Mineral compounds	Potassium (K)	6161.31 ± 794.95	5685.87 ± 1219.19	NS.
	Calcium (Ca)	252.89 ± 106.18	197.17 ± 49.86	*p* < 0.05
	Magnesium (Mg)	114.69 ± 25.99	105.34 ± 21.59	NS.
	Sodium (Na)	281.61 ± 44.43	296.59 ± 40.73	NS.
	Iron (Fe)	1.84 ± 0.58	1.94 ± 0.47	NS.
	Zinc (Zn)	1.03 ± 0.23	0.96 ± 0.12	NS.
	Copper (Cu)	0.33 ± 0.12	0.39 ± 0.11	NS.
	Manganese (Mn)	0.53 ± 0.18	0.44 ± 0.11	NS.
Vitamins	Vitamin C	64.95 ± 9.15	66.65 ± 13.32	NS.
	Vitamin B1	0.35 ± 0.18	0.37 ± 0.14	NS.
	Folates	34.52 ± 12.79	49.05 ± 12.30	*p* < 0.001

*p* < 0.05, *p* < 0.01, *p* < 0.001; determination of statistically significant differences between the variables tested; NS.: not significant; n.d.: not detected; dm: dry mass; the results are expressed as the mean values ± standard deviation of the triplicate samples; the results are expressed in the following units: carotenoids, phenolic acids, tocopherols, flavonols, mineral compounds (mg/100 g dm); vitamins: C and B1 (mg/100 g dm), folates (µg/100 g dm).

**Table 7:** Results of the correlation analysis between the contents of selected compounds with antioxidant properties in *Cucurbita pepo* L.

		Sum of				Vitamin C	Fe	Zn	Cu	Mn
		Carotenoids	Phenolic acids	Tocopherols	Flavonols					
Sum of	carotenoids	-	0.44*	0.9	0.13	0.03	−0.35	0.11	−0.47*	−0.55**
	phenolic acids	0.44*	-	−0.24	−0.33	0.42*	0.05	−0.36*	−0.13	0.06
	tocopherols	0.09	−0.24	-	−0.38	0.09	−0.48*	0.81**	–0.06	−0.20
	flavonols	0.13	−0.33	−0.38	-	–0.32	–0.15	0.05	–0.19	−0.14
Vitamin C		0.03	0.42*	0.09	−0.32	-	0.34	−0.04	−0.29	0.32
Fe		−0.35	0.05	−0.48*	−0.15	0.34	-	−0.61**	−0.20	0.61**
Zn		0.11	−0.36*	0.81**	0.05	−0.04	−0.61**	-	−0.16	−0.40*
Cu		−0.47*	−0.13	−0.06	−0.19	−0.29	−0.20	−0.16	-	0.38*
Mn		−0.55**	0.06	−0.20	–0.14	0.32	0.61**	−0.40*	0.38*	-

*p* < 0.05*; *p* < 0.01**; *p* < 0.001*** - determination of statistically significant correlations between tested variables.

**Table 8:** Results of the correlation analysis between the contents of selected compounds with antioxidant properties in *Cucurbita moschata* Duchesne.

		Sum of				Vitamin C	Fe	Zn	Cu	Mn
		Carotenoids	Phenolic acids	Tocopherols	Flavonols					
Sum of	carotenoids	-	0.47	0.08	0.91***	0.12	−0.02	−0.02	−0.22	−0.59**
	phenolic acids	0.47	-	0.41	0.37	0.01	0.63**	−0.47	−0.14	−0.08
	tocopherols	0.08	0.41	-	0.25	−0.22	0.76**	−0.09	−0.67**	0.71**
	flavonols	0.91***	0.37	0.25	-	0.23	0.08	0.13	−0.45	−0.36
Vitamin C		0.12	0.01	−0.22	0.23	-	0.12	0.15	0.01	−0.08
Fe		−0.02	0.63**	0.76**	0.08	0.12	-	−0.04	−0.61**	0.59**
Zn		−0.02	−0.47	−0.09	0.13	0.15	−0.04	-	−0.58*	0.01
Cu		−0.22	−0.14	−0.67**	−0.45	0.01	−0.61**	−0.58*	-	−0.41
Mn		−0.59**	−0.08	0.71**	−0.36	−0.08	0.59**	0.01	−0.41	-

*p* < 0.05*; *p* < 0.01**; *p* < 0.001***: determination of statistically significant correlations between tested variables.

**Table 9:** Variable levels divided into groups of pumpkin varieties.

	Compound	Cluster 1	Cluster 2	*P* Value
Carotenoids	Zeaxanthin	4.28 ± 3.97	4.87 ± 4.05	NS.
	Lutein	12.56 ± 5.96	7.47 ± 3.45	*p* < 0.01
	β-carotene	4.24 ± 2.31	3.45 ± 1.73	NS.
	Retinol equivalent	2.11 ± 0.93	1.60 ± 0.60	NS.
Phenolic acids	Gallic acid	10.97 ± 5.17	13.27 ± 7.25	NS.
	Protocatechuic acid	14.75 ± 8.47	29.19 ± 17.41	*p* < 0.001
	4-Hydroxy-benzoic acid	14.05 ± 8.36	11.94 ± 2.29	NS.
	Vanillic acid	3.95 ± 1.75	5.20 ± 2.96	NS.
	Chlorogenic acid	5.01 ±1.80	6.04 ± 1.54	NS.
	Caffeic acid	61.06 ± 36.76	46.20 ± 28.42	NS.
	P-coumaric acid	0.62 ± 0.93	0.74 ± 0.75	NS.
	Ferulic acid	15.10 ± 14.80	15.97 ± 7.68	NS.
	Sinapic acid	10.55 ± 10.64	6.89 ± 9.55	NS.
Tocopherols	α-tocopherol	4.08 ± 1.71	2.72 ± 1.20	*p* < 0.01
	γ-tocopherol	3.71 ± 2.67	3.98 ± 3.49	NS.
	δ-tocopherol	n.d.	n.d.	-
	β-tocopherol	n.d.	n.d.	-
	α-tocopherol equivalent	4.45 ± 1.83	3.12 ± 1.53	*p* < 0.05
Flavonols	Rutin	14.84 ± 13.79	11.83 ± 5.32	NS.
	Kaempferol	8.58 ± 10.30	11.78 ± 15.58	NS.
	Isoquercetin	1.23 ± 1.72	2.44 ± 2.28	NS.
	Astragalin	4.95 ± 5.95	8.28 ± 9.63	NS.
	Myricetin	2.85 ± 2.81	0.86 ± 1.37	*p* < 0.05
	Quercetin	3.15 ± 3.27	0.90 ± 1.87	*p* < 0.05
Mineral compounds	Potassium (K)	6562.65 ± 562.09	4788.11 ± 499.17	*p* < 0.001
	Calcium (Ca)	254.65 ± 100.70	182.50 ± 40.36	*p* < 0.05
	Magnesium (Mg)	113.69 ± 25.16	105.47 ± 22.99	NS.
	Sodium (Na)	294.52 ± 39.83	273.76 ± 47.54	NS.
	Iron (Fe)	1.89 ± 0.56	1.85 ± 0.50	NS.
	Zinc (Zn)	1.02 ± 0.23	0.97 ± 0.11	
	Copper (Cu)	0.35 ± 0.11	0.37 ± 0.14	NS.
	Manganese (Mn)	0.52 ± 0.17	0.44 ± 0.14	NS.
Vitamins	Vitamin C	67.64 ± 10.62	61.61 ± 10.65	NS.
	Vitamin B1	0.35 ± 0.15	0.36 ± 0.20	NS.
	Folates	35.66 ± 12.03	49.68 ± 14.50	*p* < 0.01

*p* < 0.05, *p* < 0.01, *p* < 0.001: determination of statistically significant differences between the groups of varieties; NS.: not significant; dm: dry mass; n.d.: not detected; the results are expressed as the mean values ± standard deviation of the triplicate samples; the results are expressed in the following units: carotenoids, phenolic acids, tocopherols, flavonols, mineral compounds (mg/100 g dm); vitamins: C and B1 (mg/100 g dm), folates (µg/100 g dm).
